# Two Major Medicinal Honeys Have Different Mechanisms of Bactericidal Activity

**DOI:** 10.1371/journal.pone.0017709

**Published:** 2011-03-04

**Authors:** Paulus H. S. Kwakman, Anje A. te Velde, Leonie de Boer, Christina M. J. E. Vandenbroucke-Grauls, Sebastian A. J. Zaat

**Affiliations:** 1 Department of Medical Microbiology, Center for Infection and Immunity Amsterdam (CINIMA), Academic Medical Center, University of Amsterdam, Amsterdam, The Netherlands; 2 Tytgat Institute for Liver and Intestinal Research, Academic Medical Center, University of Amsterdam, Amsterdam, The Netherlands; 3 Department of Medical Microbiology and Infectious Diseases, VU Medical Center, Amsterdam, The Netherlands; Fundació Institut Germans Trias i Pujol; Universitat Autònoma de Barcelona CibeRES, Spain

## Abstract

Honey is increasingly valued for its antibacterial activity, but knowledge regarding the mechanism of action is still incomplete. We assessed the bactericidal activity and mechanism of action of Revamil® source (RS) honey and manuka honey, the sources of two major medical-grade honeys. RS honey killed *Bacillus subtilis*, *Escherichia coli* and *Pseudomonas aeruginosa* within 2 hours, whereas manuka honey had such rapid activity only against *B. subtilis*. After 24 hours of incubation, both honeys killed all tested bacteria, including methicillin-resistant *Staphylococcus aureus*, but manuka honey retained activity up to higher dilutions than RS honey. Bee defensin-1 and H_2_O_2_ were the major factors involved in rapid bactericidal activity of RS honey. These factors were absent in manuka honey, but this honey contained 44-fold higher concentrations of methylglyoxal than RS honey. Methylglyoxal was a major bactericidal factor in manuka honey, but after neutralization of this compound manuka honey retained bactericidal activity due to several unknown factors. RS and manuka honey have highly distinct compositions of bactericidal factors, resulting in large differences in bactericidal activity.

## Introduction

Antibiotic-resistant bacteria pose a very serious threat to public health. Resistance not only is a major problem in hospitals; resistant bacteria are now recognized among various groups in the community, such as pig-breeders, and in cattle [1]–[3]. Frequencies of bacterial resistance are increasing worldwide while very few new antibiotics are being developed [4], [5]. Therefore alternative antimicrobial strategies are urgently needed.

For thousands of years honey has been used for treatment of wounds and as a gastrointestinal remedy [6], [7]. Honey has broad-spectrum activity against pathogenic and food-spoiling bacteria [8]–[11]. The potent *in vitro* activity of honey against antibiotic-resistant bacteria [8] and its successful application in treatment of chronic wound infections not responding to antibiotic therapy [12] evoked interest in honey as antibacterial agent in modern medicine. Revamil® and manuka medical-grade honey have potent antibacterial activity [9], [13] and are approved for application in wound management. RS honey, the source for Revamil®, is produced under standardized conditions in greenhouses. The factors responsible for the bactericidal activity of this honey are the high sugar concentration, H_2_O_2_, the 1,2-dicarbonyl compound methylglyoxal (MGO), the cationic antimicrobial peptide bee defensin-1 and the low pH [14].

Manuka honey is produced from the manuka bush (*Leptospermum scoparium*) indigenous to New Zealand and Australia. Exceptionally high concentrations of the antibacterial compound MGO have been found in manuka honey [15], [16], but the contribution this and possible other compounds to the bactericidal activity of manuka honey is still unknown.

Incomplete knowledge of the antibacterial factors in honey and the contribution of these factors to the bactericidal activity hamper general applicability of honey. In the current study we determined the levels of all established honey antibacterial factors in RS and manuka honey and assessed the contribution of these factors to the bactericidal activity of both honeys against food-spoiling and pathogenic bacteria, including methicillin-resistant *Staphylococcus aureus*. We demonstrate that RS and manuka honey have highly distinct compositions of bactericidal factors, resulting in substantial differences in bactericidal activity. We show that in addition to MGO several other factors contribute substantially to the bactericidal activity of manuka honey. The implications of these findings for prudent application in medicine and for the potential use of honey in food preservation are discussed.

## Methods

### Honey

Unprocessed Revamil® source (RS) honey was kindly provided by Bfactory Health Products (Rhenen, The Netherlands). Non-sterilized UMF™ 16+ Active manuka honey was purchased from Nature's nectar Limited (Surrey, UK). To study the contribution of the sugars to the bactericidal activity of honey, a solution with a sugar composition similar to that of honey was prepared (333 g/kg glucose, 385 g/kg fructose, 73 g/kg sucrose and 62 g/kg maltose).

### Microorganisms

Bactericidal activity of honey was assessed against *Bacillus subtilis* ATCC6633, Escherichia *coli* ML-35p [17], *Pseudomonas aeruginosa* PAO-1 (ATCC 15692) and against methicillin-resistant *S. aureus* (MRSA) strain AMC201 [14].

### Quantification of H2O2 in honey

H_2_O_2_ concentrations that had accumulated in diluted honey were determined quantitatively as described previously [18]. In brief, 40 µl samples of honey were added to 135 µl reagent, consisting of 50 µg/ml o-dianisidine (Sigma-Aldrich, St. Louis, MI, USA) and 20 µg/ml horseradish peroxidase type IV (Sigma-Aldrich, St. Louis, MI, USA) in 10 mM phosphate buffer pH 6.5. After 5 min. of incubation at room temperature, reactions were stopped by addition of 120 µl 6 M H_2_SO_4_ and absorption at 540 nm was measured. H_2_O_2_ concentrations were calculated using a calibration curve of 2-fold serial dilutions of H_2_O_2_ ranging from 2200 to 2.1 µM.

### Methylglyoxal (MGO) quantification and neutralization assay

Reduced glutathione (Sigma-Aldrich, St. Louis, MI, USA) was added to diluted honey to a final concentration of 15 mM, and conversion of MGO to S-D-lactoyl-glutathione (SLG) was initiated by addition of 0.5 U/ml glyoxalase I (Sigma-Aldrich, St. Louis, MI, USA). We previously determined that the bactericidal activity of a solution containing 20 mM MGO was completely neutralized by conversion to SLG [14]. The amount of MGO converted to SLG was determined using the extinction coefficient of SLG of 3.37 mM^−1^ at 240 nm [19]. As a control for complete conversion of MGO in honey, SLG formation was assessed for 40% honey solutions spiked with 10 mM of exogenous MGO.

### Analysis of bee defensin-1 in honey

Bee defensin-1 was separated from other honey bactericidal factors and analysed as described previously [14]. In brief, 15 ml of 20% (v/v) honey was centrifuged in a 5 kDa molecular weight cut-off Amicon Ultra-15 tube (Millipore, Waters, Milford, MA, USA) at 4000×g for 45 min. at room temperature. The <5 kDa filtrate was collected, and the >5 kDa retentate, where bee defensin-1 would be retained, was subsequently washed three times in the filter tube with 15 ml of demineralized water and concentrated to 0.4 ml. Duplicate samples of retentate equivalent to 150 µl of undiluted honey were subjected to native acid-urea polyacrylamide gel electrophoresis (AU-PAGE). Bee defensin-1 was subsequently visualized by parallel Coomassie-staining and by a *B. subtilis* overlay assay.

### Liquid bactericidal assay

Bactericidal activity of honey was quantified in 100 µl volume liquid tests, in polypropylene microtiterplates (Costar, Corning, NY, USA). For each experiment, a 50% (v/v) stock solution of honey was freshly prepared in incubation buffer containing 10 mM phosphate buffer pH 7.0 supplemented with 0.03% (w/v) trypticase soy broth (TSB; Difco, Detroit, MI, USA). Bacteria from logarithmic phase cultures in TSB were washed twice with incubation buffer and used at a final concentration of 1×10^6^ CFU/ml, based on optical density. Plates were incubated at 37°C on a rotary shaker at 150 rpm. At indicated time points, duplicate 10 µl aliquots of undiluted and 10-fold serially diluted incubations of three independent incubations were plated on blood agar. Bacterial survival was quantified after overnight incubation at 37°C. The detection level of this assay is 100 CFU/ml. To assess the contribution of H_2_O_2_ and cationic compounds to the bactericidal activity of honey, 600 U/ml bovine liver catalase (Sigma-Aldrich, St. Louis, MI, USA) and 0.025% (w/v) sodium polyanetholesulfonate (SPS; Sigma-Aldrich, St. Louis, MI, USA), respectively, were added to incubations. Since bee defensin-1 is the only cationic bactericidal compound present in RS honey [14], addition of SPS to this honey specifically neutralizes bee defensin-1. The incubation buffer did not affect the pH of the concentrations of honey used in our experiments. A 1 M NaOH solution was used to titrate honey solutions to pH 7.0.

### Ultrafiltration of honey components

Fifteen ml of 20% (v/v) honey was centrifuged in a 5 kDa molecular weight cut-off Amicon Ultra-15 tube (Millipore, Waters, Milford, MA, USA) at 4000×g for 45 min. at room temperature. The <5 kDa filtrate was collected, and the >5 kDa retentate was subsequently washed three times in the filter tube with 15 ml of demineralized water and concentrated to 0.4 ml.

### Bacterial overlay assay

To visualize the antibacterial activity of bee defensin-1 from honey, a bacterial overlay assay was used. Amounts of >5 kDa honey retentate equivalent to 150 µl honey were separated by acid urea polyacrylamide gel electrophoresis (AU-PAGE) [20]. Gels were either stained with PAGE-Blue (Fermentas, Vilnius, Lithuania) or washed 3×8 min. in 10 mM phosphate buffer pH 7.0 for a bacterial overlay assay. After washing, the gel was incubated for 3 hours at 37°C on *B. subtilis*-inoculated nutrient-poor agarose to allow components to diffuse into the agarose. For this agarose, a *B. subtilis* inoculum suspension was prepared as described for the liquid bactericidal assay. Bacteria (10^7^ CFU) were mixed with 20 ml nutrient-poor agar (0.03% (w/v) TSB in 10 mM sodium phosphate buffer, pH 7.0, with 1% low EEO agarose [Sigma-Aldrich, St. Louis, MI, USA]) of 45°C, and immediately poured into 10×10-cm culture plates. Subsequently, the gel was removed and the agarose was overlayed with 20 ml of double-strength nutrient agarose (6% TSB, 1% Bacto-agar, 45°C), and plates were incubated overnight at 37°C. Antibacterial activity resulting in zones devoid of bacterial growth is visualized as dark zones in a dark-field image.

## Results

### Bactericidal activity of RS and manuka honey

**Figure 1:**
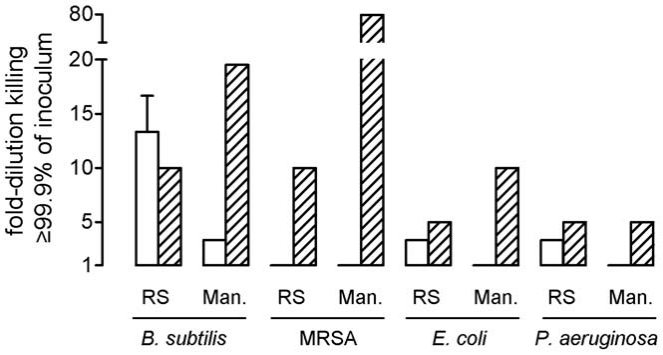
Bactericidal activity of RS and manuka honey. Indicated bacteria were incubated with serial dilutions of RS or manuka honey, starting at 40% honey. After 2 h (white bars) and 24 h (hatched bars) of incubation bacteria were quantitatively cultured. The bars represent the highest dilutions of honey causing a 1000-fold reduction in numbers of CFU relative to the initial inocula. The values are mean ± SEM of independent triplicate incubations.

The bactericidal activity of RS and manuka honey was tested against the food-spoiling bacterium *Bacillus subtilis* and against the wound pathogens methicillin-resistant *Staphylococcus aureus* (MRSA), *Escherichia coli* and *Pseudomonas aeruginosa*. We determined the maximal dilution of honey which reduced bacterial survival 1000-fold after 2 and 24 hours of incubation, using 2-fold serial dilutions of 40% (v/v) honey. After 2 h up to 13.3±3.3-fold diluted RS honey killed *B. subtilis*, whereas manuka honey could only be 2.5-fold diluted (Fig. 1). After 24 hours, RS and manuka honey had potent activity against *B. subtilis*, up to 10- and 20-fold dilution, respectively. Neither RS nor manuka honey had bactericidal activity against MRSA after 2 hours. After 24 hours RS and manuka honey did kill MRSA, at dilutions of up to 10- and 80-fold, respectively (Fig. 1). *E. coli* and *P. aeruginosa* were killed by RS honey diluted 2.5-fold at 2 hours incubation, while manuka honey lacked rapid activity against these bacteria (Fig.1). After 24 hours RS honey had bactericidal activity against both bacteria up to 5-fold dilution, and manuka honey killed *E. coli* and *P. aeruginosa* up to dilution of 10- and 5-fold, respectively (Fig. 1). Overall, RS honey clearly had more potent bactericidal activity than manuka after 2 hours of incubation, while manuka was the most potent honey after 24 hours.

### Characterization of H2O2, MGO and bee defensin-1 in RS and manuka honey

**Figure 2:**
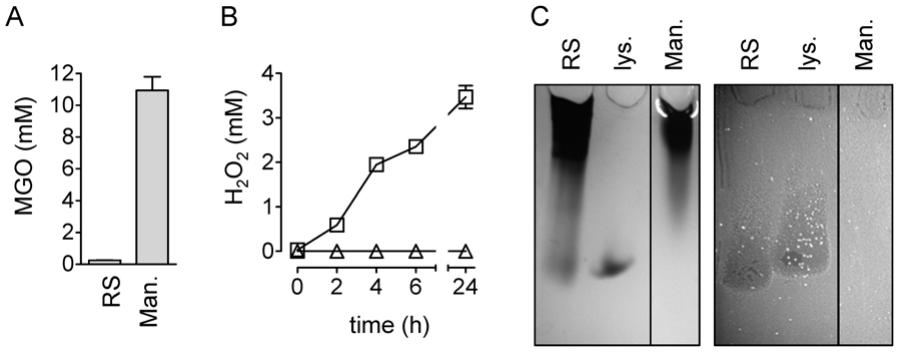
Comparison of levels of MGO, H_2_O_2_ and bee defensin-1 in RS and manuka honey. (A) Concentration of MGO in RS and manuka (Man.) honey, determined spectrophotometrically after its conversion to S-lactoylglutathione by glyoxalase I treatment. (B) H_2_O_2_ accumulation over time in 40% (v/v) RS (squares) and manuka honey (triangles). (C) Proteins were concentrated from honey by ultrafiltration with a 5 kDa molecular weight cut-off membrane. Amounts of >5 kDa retentate equivalent to 150 µl of undiluted honey, and 3 µg of lysozyme (lys.) as a reference, were run in duplicate on a single native acid-urea PAGE gel to separate cationic proteins. One half of the gel was Coomassie-stained (left), the other was used for a bacterial overlay assay with *B. subtilis* (right). Since a dark-field image was obtained, growth inhibition of the bacteria due to the presence of antibacterial proteins appears as a dark zone.

We assessed the levels of the predominant bactericidal factors in RS and manuka honey, i.e. MGO, H_2_O_2_, and bee defensin-1. RS and manuka honey contained 0.25±0.01 mM and 10.94±1.70 mM MGO, respectively (Fig. 2A). Hydrogen peroxide is produced by the *Apis mellifera* (honey bee) glucose oxidase enzyme upon dilution of honey [18], [21]. RS honey at a concentration of 40% (v/v) accumulated up to 3.47±0.25 mM H_2_O_2_ in 24 hours, while no H_2_O_2_ was detected in diluted manuka honey (Fig. 2B).

Bee defensin-1 can be visualized by parallel Coomassie-staining and a *B. subtilis* overlay assay after separation of proteins of the >5 kDa fraction of honey by native acid-urea PAGE [14]. After gel electrophoresis, bee defensin-1 in the >5 kDa retentate of RS honey produced a clear zone of growth inhibition of *B. subtilis* in a gel overlay assay (Fig. 2C). In a similar amount of manuka honey retentate no bee defensin-1 was detected either by Coomassie-staining or in a gel overlay assay (Fig. 2C). In a radial diffusion assay with *B. subtilis* an amount of RS honey retentate equivalent to 0.5 µl honey produced a zone of bacterial growth inhibition, while in an up to 20-fold larger amount of manuka retentate no antibacterial activity was observed (not shown).

### Factors contributing to the bactericidal activity of manuka honey after 24 hours

**Figure 3:**
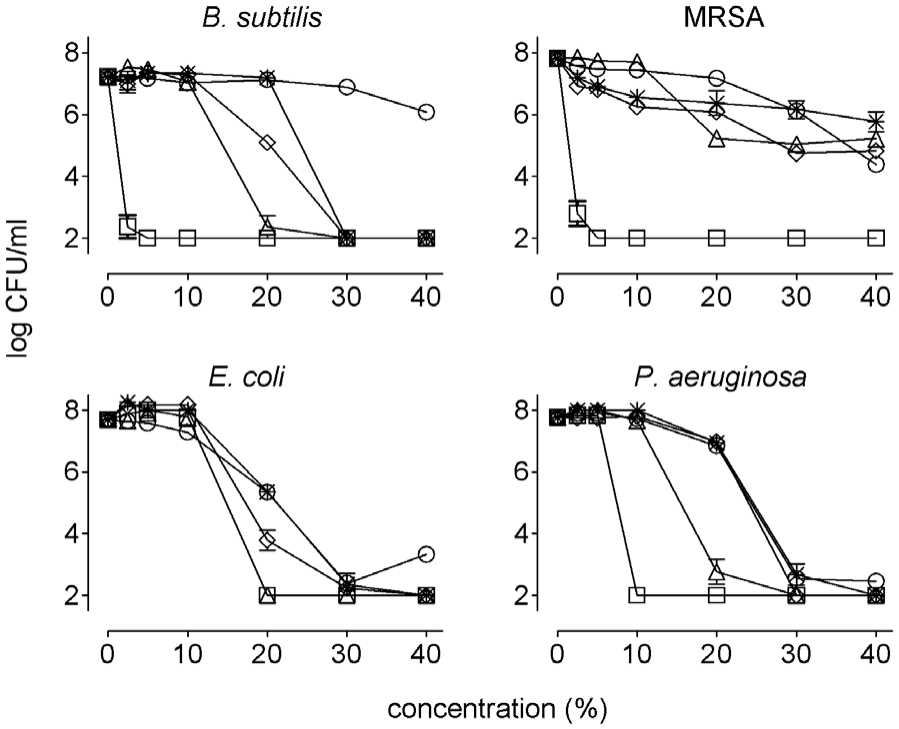
Contribution of MGO to the bactericidal activity of manuka honey. The indicated bacteria were incubated in various concentrations (v/v) of manuka honey in incubation buffer (squares), in manuka with addition of glyoxalase I (triangles) or glyoxalase I and SPS without (diamonds) or with adjustment of the pH to 7.0 (asterisks), or in a honey-equivalent sugar solution (circles). After 24 hours, numbers of surviving bacteria were determined.

We previously determined the contribution of H_2_O_2_, bee defensin-1, MGO and the low pH to the bactericidal activity of RS honey after 24 hours of incubation [14], which is summarized in Table 1. In manuka honey we did not detect H_2_O_2_ or bee defensin-1, but this honey contained an approximately 40-fold higher concentration of MGO compared to RS honey. To assess the contribution of MGO to the bactericidal activity of manuka honey after 24 hours, and to reveal potential other factors, we neutralized MGO by conversion to the non-bactericidal S-lactoylglutathione and assessed the remaining bactericidal activity. Neutralization of MGO reduced the activity against MRSA to a level identical to that of a honey-equivalent sugar solution (Fig. 3), indicating that MGO was responsible for the potent activity of manuka honey against MRSA. With MGO neutralized, 8- and 2-fold higher concentrations of manuka honey were required to kill *B. subtilis* and *P. aeruginosa*, respectively (Fig. 3). MGO-neutralized manuka honey still had more activity than a sugar solution against these species, and the activity of manuka honey against *E. coli* was not affected by neutralization of MGO (Fig. 3). This indicated the presence of other bactericidal factors beside MGO.

**Table 1:** Contribution of bactericidal factors to activity of RS and manuka honey.

	H_2_O_2_		beedefensin-1		MGO		pH		additionalcationic		additionalnon-cationic	
	RS^a^	Man	RS	Man	RS	Man	RS	Man	RS	Man	RS	Man
***B. subtilis***	rapid^b^	-	rapid	-	slow	rapid	slow	rapid	-	rapid	-	rapid
**MRSA**	slow	-	slow	-	slow	slow	-	-	-	-	-	-
***E. coli***	rapid	-	slow	-	slow	slow	-	slow	-	slow	-	slow
***P. aeruginosa***	rapid	-	slow	-	slow	slow	slow	-	-	slow	-	-

a RS: RS honey, Man: manuka honey.

b Contribution is defined as ≥1 log reduction in numbers of CFU after 2 hours (rapid) or 24 hours (slow) of incubation.

The polyanionic compound sodium polyanetholesulphonate (SPS) neutralizes cationic compounds. Addition of SPS to MGO-neutralized manuka honey abolished the residual activity against *P. aeruginosa* (Fig. 3) implying that this activity was due to cationic compound(s). As manuka honey does not contain detectable amounts of bee defensin-1, other cationic bactericidal component(s) must be present. Activity against *B. subtilis* and *E. coli* was also reduced but not abolished when SPS was added (Fig. 3), which indicates the contribution of both cationic and non-cationic factors to the non-MGO bactericidal activity of manuka honey.

When the pH of MGO- and cationic compound-neutralized manuka honey was adjusted from 3.9 to 7.0, all remaining activity against *E. coli* was abolished. Activity against *B. subtilis* was reduced, but was still substantial (Fig. 3). So, the low pH was responsible for all non-cationic bactericidal activity of MGO-neutralized manuka honey against *E. coli*, while still other non-cationic bactericidal factor(s) were involved in activity against *B. subtilis*. As expected, addition of catalase – which neutralizes H_2_O_2_ – did not affect the bactericidal activity of manuka honey (not shown), since this honey did not contain detectable levels of H_2_O_2_ (Fig 2).

In summary, the high sugar concentration, MGO, low pH and as yet unidentified cationic factor(s) and non-cationic bactericidal factor(s) contributed to the bactericidal activity of manuka honey as recorded after 24 h.

### Factors contributing to the rapid bactericidal activity of RS and manuka honey

**Figure 4:**
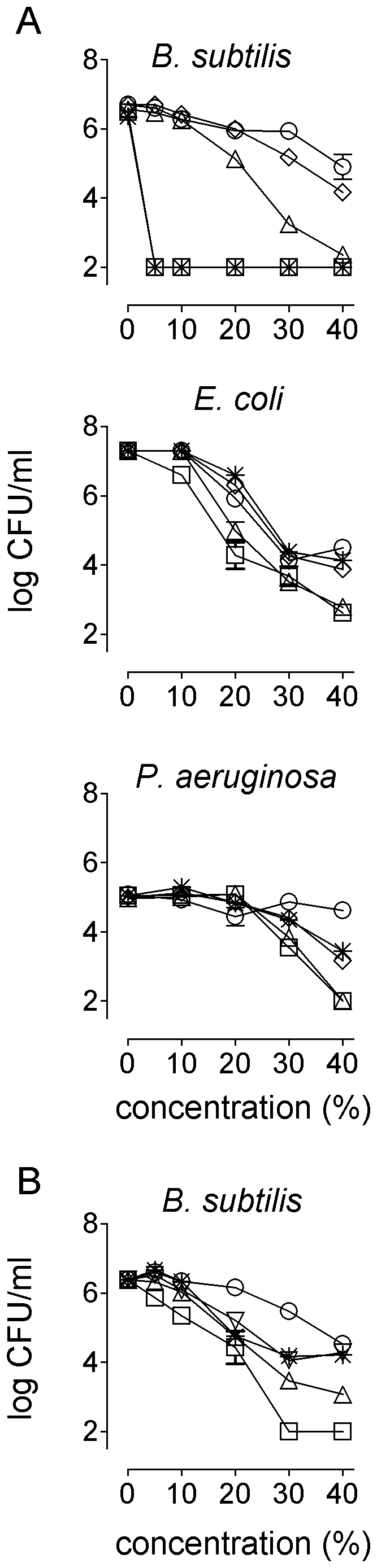
Factors contributing to rapid bactericidal activity of RS and manuka honey. (A) RS honey; bacteria were incubated for 2 hours in various concentrations (v/v) of RS honey in incubation buffer (squares), with either catalase (asterisks), SPS (triangles) or both (diamonds) added, or in sugar solution (circles). (B) Manuka honey; *B. subtilis* was incubated for 2 hours in various concentrations of manuka honey in incubation buffer (squares), with successive addition of glyoxalase I (triangles, top up), SPS (triangles, top down), and titration to pH 7.0 (asterisks), or in sugar solution (circles).

In figure 1 we showed that RS honey had rapid activity (within 2 hours) against *B. subtilis*, *E. coli* and *P. aeruginosa* while manuka honey exerted rapid activity only against *B. subtilis*. We subsequently assessed the contribution of individual factors to this rapid bactericidal activity. The entire *B. subtilis* inoculum was killed within 2 hours by 5% RS honey (Fig. 4A). When bee defensin-1 was neutralized by addition of SPS, 40% RS honey was required to kill *B. subtilis*. Subsequent neutralization of H_2_O_2_ reduced the activity of RS honey to that of a honey-equivalent sugar solution (Fig. 4A). So, bee defensin-1 and to a lesser extent H_2_O_2_, were the major factors involved in rapid activity of RS honey against *B. subtilis*.

Neutralization of bee defensin-1 did not affect the activity of RS honey against *E. coli* or *P. aeruginosa* (Fig. 4A). After neutralization of H_2_O_2_ the activity of RS honey against *E. coli* was reduced to that of a sugar solution and the activity of 40% RS honey against *P. aeruginosa* was reduced to a level at which the numbers of CFU were only about 1-log lower than after incubation in sugar (Fig. 4A). Titration of honey to pH 7 abolished all residual activity against *P. aeruginosa* (not shown). So, in different combinations, bee defensin, H_2_O_2_, sugars and the low pH contributed to the rapid activity of RS honey against specific bacterial species.

Concentrations of ≥30% manuka honey were required for rapid activity against *B. subtilis* (Fig. 4B). Neutralization of MGO in manuka honey reduced but did not abolish this rapid activity (Fig. 4B). The remaining activity was not affected by titration to pH 7 (Fig. 4B) or addition of catalase (not shown), but this activity was further reduced but not abolished by addition of SPS (Fig. 4B). Both the cationic and non-cationic factor(s) involved in this rapid activity are unknown.

## Discussion

General applicability of honey as antimicrobial agent requires safe preparations, knowledge of the composition of antibacterial factors and standardized antibacterial activity. Medical-grade honeys are gamma-irradiated to eradicate bacterial spores which may be present in raw honey. Revamil® and manuka medical-grade honey are approved as a medical product for wound care. Manuka honey obtained from manuka trees (*Leptospermum scoparium*) in New Zealand and Australia has large batch-to-batch variation in antibacterial activity [13]. Medical-grade manuka honey often carries a UMF™ (Unique Manuka Factor) value representing its antimicrobial activity. This value is based on activity against *S. aureus* in an agar diffusion assay. Standardization of RS honey, the source for Revamil®, is based on a controlled production process in greenhouses. We have previously shown that the bactericidal activity of Revamil® honey against *B. subtilis* varies by less than a factor two for eleven batches of honey [9], but the antibacterial activity of this honey is not routinely assessed.

In medicine, honey may be used either for treatment of infection or as antibacterial prophylaxis. Honey applied to wounds is diluted by wound exudate, so the active compounds need to be present in high concentrations. Treatment of infections particularly requires rapid bactericidal activity, whereas prophylactic application demands sustained and not necessarily very rapid bactericidal activity. We therefore assessed the rapid and slow bactericidal activity of RS and manuka honey, i.e. the activity after 2 and 24 hours of incubation, respectively. RS honey had much more potent rapid activity than manuka honey against *B. subtilis*, *E. coli* and *P. aeruginosa*. Both RS and manuka honey lacked rapid activity against MRSA. With respect to slow bactericidal activity, manuka honey was more potent than RS honey, most notably against MRSA and *B. subtilis*.

RS honey contains relatively high levels of bee defensin-1 and H_2_O_2_ and only a minor amount of MGO, whereas the opposite is true for manuka honey. The contribution of these compounds for rapid and slow bactericidal activity of RS and manuka honey is summarized in Table 1. The main conclusion is that these honeys exert bactericidal activity through entirely different sets of compounds, resulting in distinct bactericidal properties. MGO contributed substantially to the activity of manuka honey against *S. aureus* and *B. subtilis* but not against *E. coli* and *P. aeruginosa*. The activity against these latter bacteria involved compounds other than MGO including as yet unidentified cationic and non-cationic compounds. In earlier studies before the discovery of MGO, acidic [22] and phenolic compounds [23], [24] were isolated from manuka honey. The contribution of these factors to the bactericidal activity was questioned at that time, since their concentrations in manuka honey were far too low to account for all observed activity [25]. It is possible that acidic and phenolic compounds are responsible for the non-MGO bactericidal activity of manuka honey. Bee defensin-1 and H_2_O_2_ were responsible for most of the rapid activity of RS honey and the absence of these compounds explains the limited rapid bactericidal activity of manuka honey.

Honeys show wide variation in their capacity to produce H_2_O_2_; some honeys – including the manuka honey used in our study - do not accumulate H_2_O_2_ at all [18], [26]. Several causes for the absence of H_2_O_2_ in honey have been proposed. Factors known to affect H_2_O_2_ accumulation are the inactivation of glucose oxidase due to exposure to excess heat or light [27], [28] degradation of H_2_O_2_ by catalase originating from nectar [29], and chemical scavenging [30]. Another explanation for the variation in H_2_O_2_ accumulation in honeys could be differences in levels of glucose oxidase. To our knowledge no studies have been performed to assess the concentration of glucose oxidase in honey.

Bee defensin-1 is secreted in honey by the honey bee hypopharyngeal gland [14], but we did not detect bee defensin-1 in manuka honey. Secretions of the hypopharyngeal gland are used for production of royal jelly and honey [31], [32]. The amount of bee defensin-1 in royal jellies (therein referred to as ‘royalisin’) obtained from different apiaries varies strongly [33], with some samples completely devoid of this peptide. This implies that bee defensin-1 expression in hypopharyngeal glands and/or the amount of gland secretions added may vary strongly. This could also explain the difference in bee defensin-1 levels in RS and manuka honey.

Recently, the 1,2-dicarbonyl compound MGO was identified as antibacterial compound present in exceptionally high levels in manuka honey [15], [16]. In general, MGO can be formed from sugars during heat-treatment or prolonged storage of carbohydrate-containing foods and beverages [34]. MGO in manuka honey however is formed by conversion of dihydroxyacetone (DHA) present at exceptionally high concentrations in the nectar of manuka trees (*Leptospermum scoparium*) [35]. It is unknown how DHA is formed in nectar and why it is present in such large amounts in manuka trees. Concentrations of MGO in fermented milk products, wine, beer and roasted coffee have been reported to be in the range of 3 to 47 mg/kg, while up to 828 mg/kg (16.1 mM), has been found in manuka honey [15], [16]. MGO is a reactive metabolite that can exert toxic effects [36]. Manuka honey has a long history of safe use, but nowadays batches of manuka honey with maximized levels of MGO are selected for medical and nutritional use. Concerns regarding potential toxicity of dietary MGO in honey and the effects on wound healing have been expressed by others [16], [37] and this remains to be investigated.

The antibacterial properties of honey, or of individual honey components, could also be interesting for application in food technology, e.g. for food preservation or as functional food ingredients. It has for instance been reported that honey can inhibit opportunistic bacterial growth in milk [38]. We show that the food spoilage bacterium *B. subtilis* is highly susceptible to manuka honey, and also *Bacillus cereus* is effectively killed by this honey (data not shown). Since manuka honey retains bactericidal activity against food-spoiling bacilli up to very high dilution, this honey has better potential than RS honey for food preservation.

Lack of standardization of antibacterial activity and incomplete knowledge of the active components are major limitations for the application of honey in medicine. The antibacterial activity of medical-grade manuka honey is commonly assessed by an agar diffusion assay with *S. aureus* [13]. This method has several major limitations. Firstly, antibacterial activity against a single bacterial species is not representative for activity against other species, since different species have varying susceptibility to honey and its antibacterial factors. We show for instance, that *E. coli* and *P. aeruginosa* are substantially less susceptible to manuka honey than *S. aureus* and *B. subtilis*. Secondly, in the agar diffusion assay the activity of honey is estimated by the size of the growth inhibition zone. Obviously, the size of such zones not only depends on the antimicrobial activity, but also on the rate of diffusion of antibacterial factors through the agar matrix. Honey with potent antibacterial activity due to compounds with relatively high molecular weight may thus erroneously be characterized as having low activity. Thirdly, the agar diffusion test does not discriminate between growth inhibiting and bactericidal activity and does not allow quantification of bactericidal activity or kinetics of killing.

To assess the potential of honey for treatment of infection it is important to discriminate between bacteriostatic and bactericidal activity, and to quantify the latter activity. This is also highly relevant for application of honey or honey-derived components in food preservation. In the current study, these limitations were overcome by the use of a quantitative liquid bactericidal assay with a panel of representative bacterial species.

Detailed analysis of antibacterial factors and bactericidal activity against a representative panel of bacteria is essential to characterize honeys. Such characterization will allow the production of standardized honeys with defined antibacterial activity, contributing to their applicability for medical, nutritional and food preservation purposes.
